# Blood-Based Analysis of Different Tau Variants in Patients With Multiple Traumatic Injuries

**DOI:** 10.1001/jamanetworkopen.2025.58573

**Published:** 2026-02-10

**Authors:** Rebecca Halbgebauer, Fernando Gonzalez-Ortiz, Benjamin Mayer, Claudius Berger, Christian Bergmann, Helen Rinderknecht, Eberhard Barth, Lisa Wohlgemuth, Marco Mannes, Markus Otto, Hayrettin Tumani, Borna Relja, Florian Gebhard, Markus Huber-Lang, Henrik Zetterberg, Steffen Halbgebauer, Kaj Blennow

**Affiliations:** 1 Institute of Clinical and Experimental Trauma Immunology, Ulm University Medical Center, Ulm, Germany; 2 Department of Psychiatry and Neurochemistry, University of Gothenburg, Mölndal, Sweden; 3 Institute for Epidemiology and Medical Biometry, Ulm University, Ulm, Germany; 4 Translational and Experimental Trauma Research, Department of Trauma, Hand, and Reconstructive Surgery, Ulm University Medical Center, Ulm, Germany; 5 Department of Anesthesiology, Ulm University Medical Center, Ulm, Germany; 6 Department of Neurology, University Hospital Halle, Halle (Saale), Germany; 7 Department of Neurology, Ulm University Hospital, Ulm, Germany; 8 Clinical Neurochemistry Laboratory, Sahlgrenska University Hospital, Mölndal, Sweden; 9 Department of Neurodegenerative Disease, UCL Institute of Neurology, University College London, London, United Kingdom; 10 UK Dementia Research Institute at University College London, London, United Kingdom; 11 Hong Kong Center for Neurodegenerative Diseases, InnoHK, Hong Kong, China; 12 Wisconsin Alzheimer’s Disease Research Center, University of Wisconsin School of Medicine and Public Health, University of Wisconsin, Madison; 13 German Center for Neurodegenerative Diseases, Ulm, Germany; 14 Paris Brain Institute, Institut du Cerveau et de la Moelle Epinière, Pitié-Salpêtrière Hospital, Sorbonne University, Paris, France; 15 Neurodegenerative Disorder Research Center, Division of Life Sciences and Medicine, and Department of Neurology, Institute on Aging and Brain Disorders, University of Science and Technology of China and First Affiliated Hospital of University of Science and Technology of China, Hefei, P.R. China

## Abstract

**Importance:**

With blood-based phosphorylated tau biomarkers soon to be used for diagnosis of Alzheimer disease, analyzing tau levels in other conditions could enhance biomarker interpretability. Moreover, mechanisms of tau release into circulation remain unclear.

**Objective:**

To evaluate concentrations of phosphorylated and nonphosphorylated tau variants in the blood of patients with multiple traumatic injuries on days 0, 1, 5, and 10 and investigate biological processes driving tau release.

**Design, Setting, and Participants:**

This multiple-trauma cohort (injury severity score, ≥18) included 45 severely injured patients with (n = 27) and without (n = 18) moderate-to-severe traumatic brain injury on emergency computed tomographic imaging. Controls consisted of 24 healthy volunteers. Participants were recruited from December 1, 2013, to October 31, 2022. Blood samples were analyzed for brain-derived tau (BD-tau), total tau (t-tau), and phosphorylated tau 217 (p-tau217) and 231 (p-tau231) levels. Associations among tau concentrations, clinical data, and outcome (eg, Glasgow Coma Scale [GCS] score) were assessed. Data were analyzed from March 1, 2023, to September 30, 2024.

**Exposures:**

Serum BD-tau, t-tau, p-tau217, and p-tau231 levels.

**Results:**

A total of 214 serum samples were analyzed. Median age of the 45 patients was 48 (IQR, 33-60) years (35 [77.8%] male); median age of the 24 controls, 43 (IQR, 28-50) years (16 [66.7%] male). Median serum levels of tau variants were increased in patients with multiple traumatic injuries at day 0 compared with controls (t-tau: 43 [IQR, 21-95] vs 3 [IQR, 3-5] pg/mL; BD-tau: 78 [IQR, 30-343] vs 2 [IQR, 2-3] pg/mL; p-tau231: 61 [IQR, 21-79] vs 2 [IQR, 1-3] pg/mL; all, *P* < .001). Only median BD-tau levels remained elevated until day 10 (day 1, 25 [IQR, 14-69] pg/mL; day 5, 9 [IQR, 4-15] pg/mL; day 10, 8 [IQR, 4-18] pg/mL). Median tau levels at admission were higher in patients with lower GCS scores (BD-tau: 107 [ IQR, 59-838] vs 33 [IQR, 24-78] pg/mL [*P* = .01]; p-tau231: 76 [IQR, 36-114] vs 28 [IQR, 9–63] pg/mL [*P* = .02]). Elevated median tau levels were also observed in patients with hemorrhagic shock vs those without shock (eg, BD-tau on day 0: 113 [IQR, 78-378] vs 31 [IQR, 24-61] pg/mL; *P* = .002) and in nonsurvivors vs survivors with uncomplicated courses (eg, BD-tau on day 1: 92 [IQR, 22-527] vs 16 [IQR, 7-23] pg/mL; *P* = .009).

**Conclusions and Relevance:**

In this exploratory study among a cohort of patients with multiple traumatic injuries, levels of tau variants reflected both direct and indirect neurological injury, with BD-tau showing the most persistent elevation in the acute phase.

## Key Points

**Question:**

Are blood-based tau biomarkers clinically useful to monitor neurological injuries in patients with multiple traumatic injuries?

**Findings:**

In this cohort study with 69 participants, total, phosphorylated, and brain-derived tau blood levels were significantly elevated in patients with multiple traumatic injuries, with and without head injury. Tau concentrations were associated with injury severity, shock, and clinical outcome.

**Meaning:**

These findings suggest that further research may inform on the usefulness of blood-based tau biomarkers for monitoring neurological damage and their association with clinical outcomes.

## Teaser

This cohort study evaluates whether there are differences in blood-based phosphorylated and nonphosphorylated tau variant levels in patients with multiple traumatic injuries and healthy controls.

## Introduction

The tau protein has largely been studied due to its major implications in neurodegenerative conditions, mainly in Alzheimer disease (AD).^1,2^ As a microtubule-associated protein, tau plays a vital role in maintaining neuronal stability and regulating intracellular transport.^3,4^ However, its importance extends beyond its conventional function, with multiple forms exhibiting distinct roles in various physiological processes not only in the central nervous system but also in peripheral organs.^5,6,7,8,9^

Under pathological conditions, tau can undergo abnormal posttranslational modifications, particularly phosphorylation.^10^ Hyperphosphorylation leads to the detachment of tau from microtubules, causing their destabilization and subsequent aggregation into neurofibrillary tangles.^11^ Despite their strong association with AD, phosphorylated and nonphosphorylated forms of tau have been reported to be increased in different conditions in the absence of amyloid deposition.^9,12^ In the context of acute neuronal injury, increased levels of different tau species have been associated with severity and clinical outcome, suggesting clinical potential as useful markers beyond AD.^13,14,15,16^

Several mechanisms may contribute to the increased levels of phosphorylated and nonphosphorylated tau species in blood following acute neuronal injury. Mechanical disruption of neuronal membranes and axonal structures, such as that occurring after traumatic brain injury (TBI) or ischemic stroke, can lead to the passive release of cytosolic proteins, including tau, into the extracellular space and ultimately into circulation.^4^ In parallel, acute injury induces excitotoxicity, oxidative stress, and calcium dysregulation, all of which activate intracellular kinases (eg, glycogen synthase kinase 3 β, cyclin-dependent kinase 5, and mitogen-activated protein kinase) that promote tau phosphorylation and detachment from microtubules.^17^ Inflammatory responses and blood-brain barrier (BBB) disruption further facilitate the leakage of tau species from the central nervous system into the bloodstream.^17^ Together, these processes likely interact to shape the complex profile of tau alterations observed in the blood during acute neuronal injury.

Plasma measurements of different tau variants represent promising diagnostic and monitoring tools for acute neurological conditions.^18,19^ With the imminent implementation of plasma phosphorylated tau (p-tau) assessment into clinical practice for AD screening and diagnosis, the evaluation of these markers in different conditions could add significant benefits to the portfolio of markers in acute disorders, such as TBI and stroke.^1^ Moreover, there is limited knowledge on the primary factors that trigger the release of tau into circulation, particularly in acute scenarios.

Patients with multiple traumatic injuries present a complex clinical picture characterized by multiple concurrent injuries that affect various organ systems, leading to intricate pathophysiological interactions and a high risk of complications.^20,21^ Effective management requires a multidisciplinary approach to address the dynamic and multifaceted nature of their condition. This includes the timely and effective diagnosis, monitoring, and treatment of neurological damage, such as that seen after TBI.^22^ In this context, blood-based biomarkers such as tau offer a practical and scalable approach for rapid assessment of neuronal injury, especially in emergency or resource-limited settings. Their integration into clinical workflows could improve triage accuracy, enable early intervention, and ultimately help reduce the societal and health care burden associated with TBI.

In this study, we conducted a differential evaluation of specific p-tau and non–p-tau variants in the blood of severely injured patients in the acute and subacute phases after multiple injuries. We then sought the underlying mechanisms determining the extent of tau release into the blood.

## Methods

### Clinical Study

A monocenter, controlled, longitudinal cohort study with prospective sample collection was conducted according to the Declaration of Helsinki and its modifications.^23^ The study protocol was approved by the Local Ethics Committee of Ulm University, and the study is ongoing. Study participants were recruited from December 1, 2013, to October 31, 2022, at Ulm University Hospital (a level I trauma care center) after informed written consent; if patients were unconscious at the time of hospital admission, consent was obtained after they regained consciousness or from their legal representatives. We followed the Strengthening the Reporting of Observational Studies in Epidemiology (STROBE) reporting guideline.

Serum samples were collected on hospital admission and 1, 5, and 10 days after admission. Further detailed patient information is found in eMethods and eTable 1 in Supplement 1. Potential participants for the healthy volunteer group (n = 24) were recruited at Ulm University by internal communication channels such as staff newsletters, bulletin boards, and institutional mailing lists. None of the healthy volunteers were reported to have a history of any neurological condition.

### Serum Biomarker Measurements

Serum brain-derived tau (BD-tau), p-tau231, and p-tau217 measurements were performed on a bead-based immunoassay platform (Simoa HD-X Analyzer; Quanterix) with a method previously described.^24,25^ eMethods in Supplement 1 provides detailed information on assays used.

### Statistical Analysis

Data were analyzed from March 1, 2023, to September 30, 2024. Data are shown as individual values with medians and IQRs. Statistical analysis was performed using Prism, version 10.4.0 (GraphPad Software) and R, version 4.5.0 (R Project for Statistical Computing). Two-sided *P* < .05 indicated statistical significance. eMethods in Supplement 1 provides a detailed description of all statistical analyses used.

## Results

### Study Participants

In total, 69 participants were included in the study. Of those, 45 were patients with severe injury and 24 were healthy volunteers. A total of 214 serum samples were analyzed. The median age of patients was 48 (IQR, 33-60) years, with 10 (22.2%) identified as female and 35 (77.8%) as male. In the control group, the median age was 43 (IQR, 28-50) years, with 8 (33.3%) identified as female and 16 (66.7%) as male. There were no significant differences regarding age or sex distribution between patients with multiple traumatic injuries and healthy controls (further details and association of biomarkers with age and of sex are found in eFigures 1 and 2 and eTables 2 and 3 in Supplement 1). Demographic features and clinical data of patients and healthy volunteers are summarized in the Table.

**Table.** Demographic Features and Clinical Data of Patients and Healthy Controls

Characteristic	Overall study cohort			Cohort with multiple traumatic injuries only		
	Healthy controls (n = 24)	Patients with multiple traumatic injuries	*P* value	Non-TBI (n = 18)	TBI (n = 27)	*P* value
Sex, No. (%)^a^						
Female	8 (33.3)	10 (22.2)	.39	7 (38.9)	3 (11.1)	.06
Male	16 (66.7)	35 (77.8)		11 (61.1)	24 (88.9)	
Age, median (IQR), y^b^	43 (28 to 50)	48 (33 to 62)	.09	57 (27 to 64)	46 (34 to 59)	.37
ISS score, median (IQR)^c^	NA	27 (22 to 34)	NA	27 (20 to 34)	27 (22 to 34)	.58
GCS score, median (IQR)^d^	NA	12 (7 to 15)	NA	15 (12 to 15)	8.5 (3 to 15)	.008^e^
AIS score head, median (IQR)^f^	NA	2 (0 to 3.5)	NA	0 (0 to 0)	3 (2 to 5)	<.001^e^
Base excess, median (IQR), mEq/L	NA	−2.8 (−4.8 to −0.9)	NA	−2.9 (−6.6 to −0.93)	−2.7 (−4.6 to −0.9)	.52

Abbreviations: AIS, Abbreviated Injury Scale; GCS, Glasgow Coma Scale; ISS, Injury Severity Scale; NA, not applicable; TBI, traumatic brain injury.

SI conversion factor: To convert base excess to mmol/L, multiply by 1.

^a^ Compared using Fisher exact test.

^b^ Compared using Mann-Whitney test.

^c^ Scores range from 1 to 75, with higher scores indicating greater severity of injury.

^d^ Scores range from 1 to 15, with higher scores indicating greater consciousness.

^e^ Indicates statistical significance.

^f^ Scores range from 0 to 6, with higher scores indicating greater severity of injury.

### Circulating Total Tau and BD-Tau After Severe Multiple Injuries

**Figure 1.**
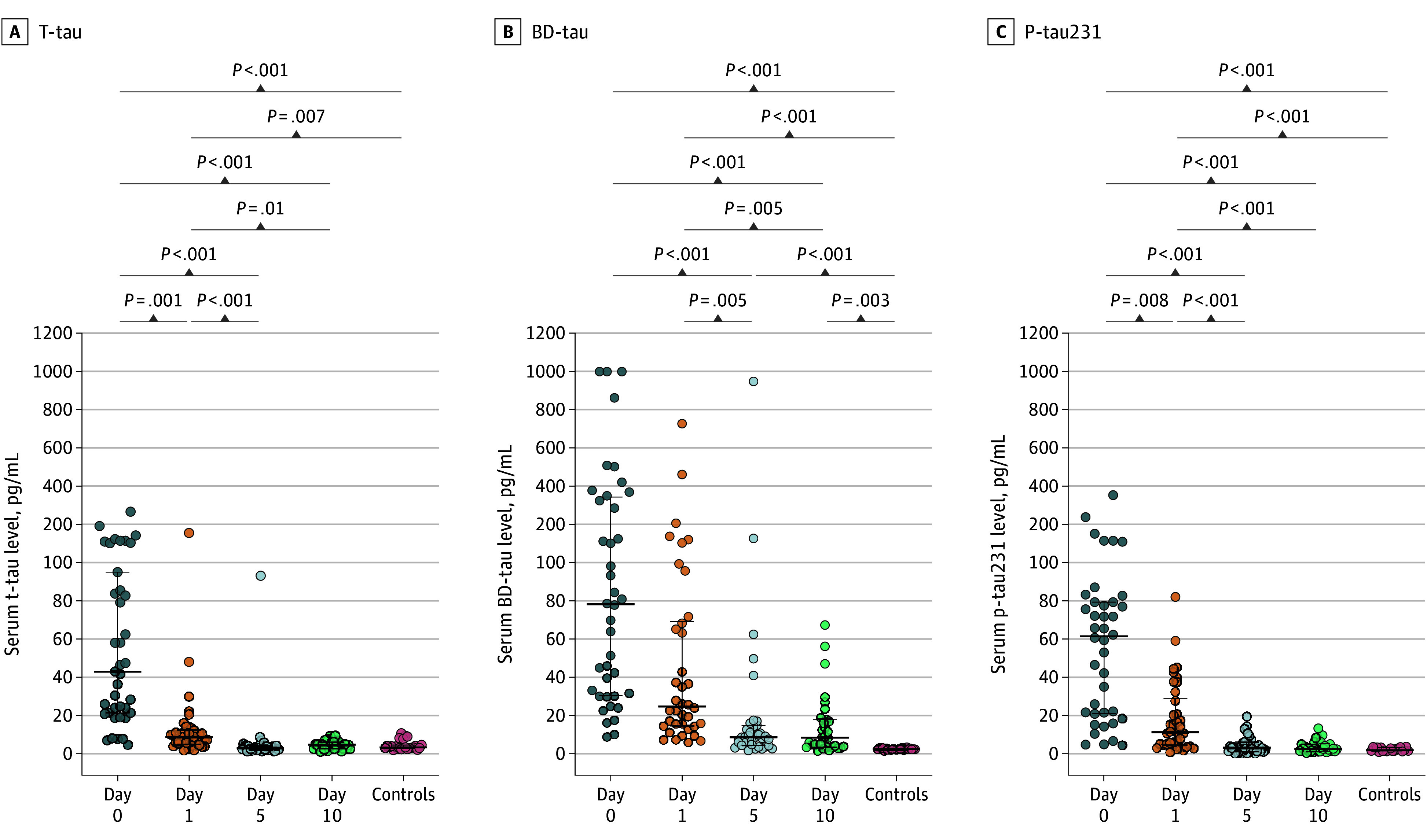
Tau Trajectories in Patients With Multiple Injuries Serum concentrations of total tau (t-tau), brain-derived tau (BD-tau), and phosphorylated tau 231 (p-tau231) were measured in patients with multiple traumatic injuries on arrival at the emergency department (day 0) and at the designated time points after injury as well as in healthy controls. Note that the levels of the tau variants are not directly comparable in numerical sense due to differences in assay design and the use of distinct calibrators and standard curves.

We assessed tau variants during the posttraumatic course compared with healthy controls and found significantly elevated concentrations of serum total tau (t-tau) (Figure 1A) and BD-tau (Figure 1B), especially during the first days after admission (day 0 t-tau: 43 [IQR, 21-95] vs 3 [IQR, 3-5] pg/mL; day 0 BD-tau: 78 [IQR, 30-343] vs 2 [IQR, 2-3] pg/mL; day 1 t-tau: 9 [IQR, 5-13] vs 3 [IQR, 3-5] pg/mL; day 1 BD-tau: 25 [IQR, 14-69] vs 2 [IQR, 2-3] pg/mL). p-Tau231 levels were significantly elevated at day 0 (61 [IQR, 21-79] vs 2 [IQR, 1-3] pg/mL) but decreased by day 1 (11 [IQR, 5-29] vs 2 [IQR, 1-3] pg/mL). At 5 and 10 days of follow-up, BD-tau (day 5, 9 [IQR, 4-15] pg/mL; day 10, 8 [IQR, 4-18] pg/mL) but not t-tau (day 5, 3 [IQR, 2-4] pg/mL; day 10, 5 [IQR, 3-6] pg/mL) or p-tau231 (day 5, 3 [IQR, 1-6] pg/mL; day 10, 5 [IQR, 3-6] pg/mL) still displayed significantly increased levels compared with the controls (eTable 4 in Supplement 1). After adjusting for the important confounding factors age, sex, and repeated measures by using linear mixed models and estimating confounder-adjusted marginal means, we observed a significant difference on day 0 between t-tau (estimated margin mean, 64.62 [SE, 5.90] pg/mL; *P* < .05) and BD-tau (estimated marginal mean, 187.33 [SE, 28.60] pg/mL; *P* < .05) in patients compared with healthy controls (eTables 5 and 6 in Supplement 1).

### P-Tau Variants in Blood After Severe Multiple Injuries

In our patient cohort, we detected significantly increased p-tau231 levels as noted above (Figure 1C), especially early after hospital admission, returning to baseline within 5 days. This increase remained significant when comparing patients on day 0 with controls after adjusting for age, sex, and repeated measures (estimated marginal mean, 59.80 [SE, 6.50] [pg/mL]; *P* < .05) (eTable 7 in Supplement 1). The same trajectory was also observed for p-tau217, with significantly higher serum concentrations during the first 24 hours after injury in a smaller subcohort (eFigure 3 in Supplement 1). As early as hospital admission after trauma, there was a strong correlation between all tau variants (Spearman *r* range, 0.80-0.93), which decreased considerably during the posttraumatic course until day 10 (Spearman *r* range, 0.49-0.56) (heat maps and 95% CIs of Spearman *r* coefficients are found in eFigure 4 in Supplement 1).

### Tau Blood Levels and (Neuronal) Injury Severity

**Figure 2.**
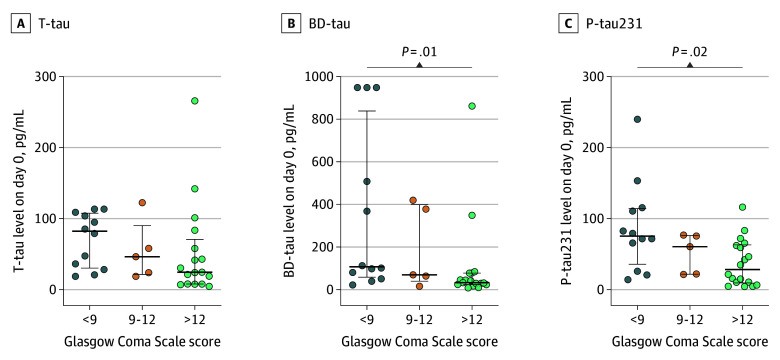
Tau Levels Stratified by Glasgow Coma Scale Score Serum concentrations of total tau (t-tau), brain-derived tau (BD-tau), and phosphorylated tau 231 (p-tau231) at day 0 were stratified according to Glasgow Coma Scale stages (<9 indicates severe; 9-12, moderate; and >12, mild).

When grouping patients for their neurological function, that is, the GCS score determined by the attending emergency physician at the injury scene, we found significant intergroup differences (severe vs mild TBI), especially for BD-tau (107 [ IQR, 59-838] vs 33 [IQR, 24-78] pg/mL; *P* = .01) and p-tau231 (76 [IQR, 36-114] vs 28 [IQR, 9–63] pg/mL; *P* = .02) among patients with lower GCS scores, but not for t-tau (82 [IQR, 30-108] vs 25 [IQR, 8-71] pg/mL; *P* = .19) (Figure 2). This finding was confirmed by a lack of significant differences in tau concentrations between patients with vs without severe head injury. For this analysis, we found that allocating patients to a cohort with no or only minor head injury (Abbreviated Injury Scale [AIS] score of the head 0-1) or with significant head injury (AIS score of ≥2) based on whole-body emergency computed tomographic scans (eFigure 5 in Supplement 1) did not result in intergroup differences in serum tau concentrations at any time point after injury. To unravel potential differences between patients with and without TBI masked by important confounders, we also adjusted results for age and sex, but did not observe any significant differences in estimated marginal means (eTables 8-13 in Supplement 1). Nevertheless, whole-body injury severity was associated with the amount of tau proteins found in serum after trauma, with an overall Injury Severity Score of more than 32 (ie, highly severe injury) associated with significantly higher serum tau concentrations—mostly irrespective of the isoform—during the early phase post injury (eFigure 5 in Supplement 1).

### Hemorrhagic Shock and Blood Tau Levels

**Figure 3.**
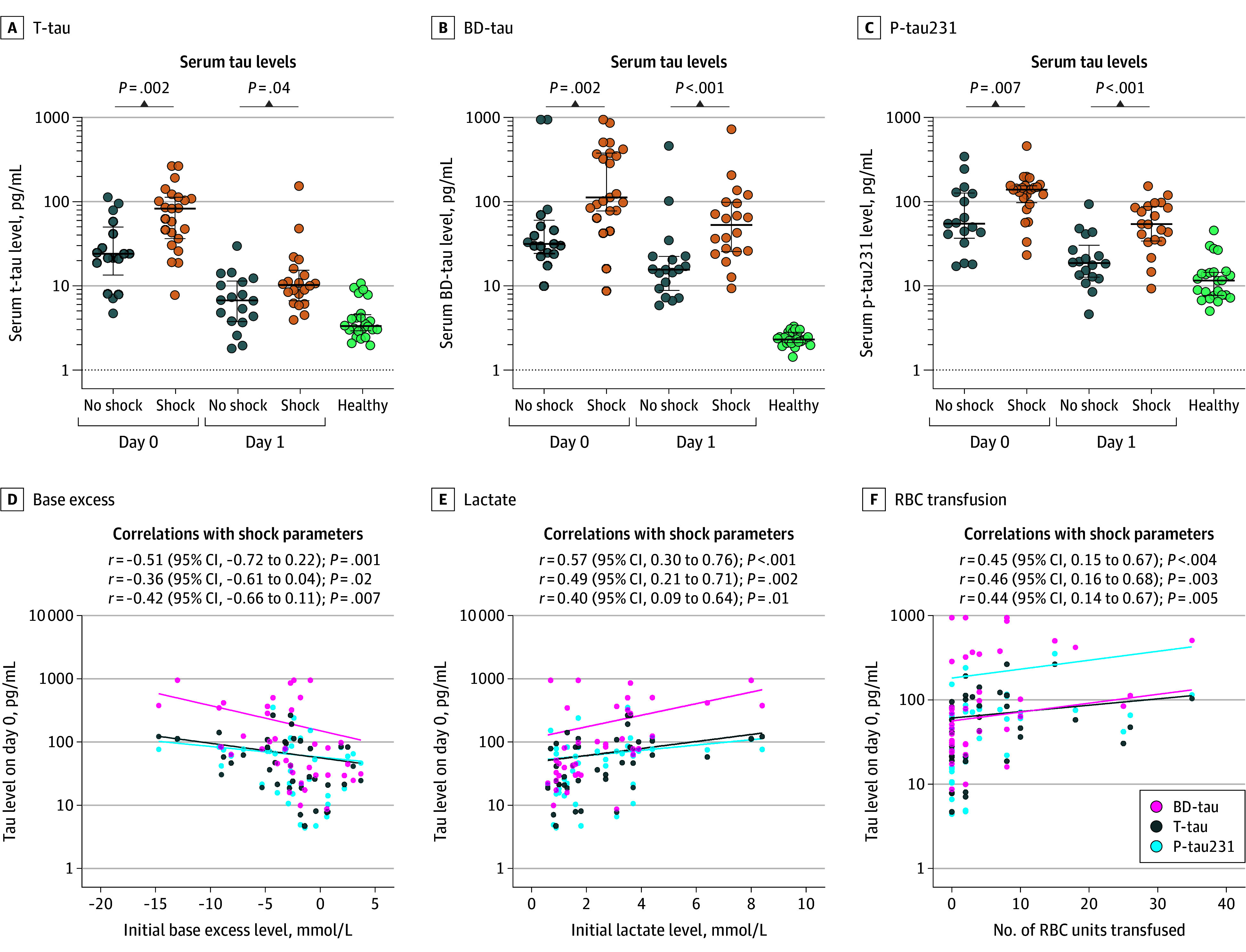
Tau Isoform Concentrations in Association With Hemorrhagic Shock Serum concentrations of total tau (t-tau), brain-derived tau (BD-tau), and phosphorylated tau 231 (p-tau231) were stratified by shock. All 3 variants showed significantly increased serum concentrations on days 0 and 1 in patients with shock. RBC indicates red blood cell.

Since blood loss and shock frequently result in tissue hypoxia and possibly subsequent induction of neurodegenerative processes, we assessed tau blood levels depending on the presence or absence of severe hemorrhage according to the established parameters blood base excess, lactate, and the number of RBC units transfused during the first 24 hours after admission. Compared with patients without shock, we found significantly higher serum levels of t-tau in patients with shock (day 0: 83 [IQR, 36-114] vs 24 [IQR, 13-50] pg/mL [*P* = .002]; day 1, 10 [IQR, 7-15] vs 7 [IQR, 4-11] pg/mL [*P* = .04]), BD-tau (day 0: 113 [IQR, 78-378] vs 31 [IQR, 24-61] pg/mL [*P* = .002]; day 1: 3 [IQR, 26-98] vs 16 [IQR, 9-23] pg/mL [*P* < .001]), and p-tau231 (day 0: 72 [IQR, 45-83] vs 21 [IQR, 12-64] pg/mL [*P* = .007]; day 1: 20 [IQR, 11-39] vs 5 [IQR, 3-10] pg/mL [*P* < .001]) (Figure 3A-C). Moreover, we observed a correlation of initial (ie, emergency department) tau values with shock parameters of initial base excess, blood lactate levels, and the number of transfused RBC units during the first 24 hours (Figure 3D-F), which mostly persisted until day 1 (eFigure 6 in Supplement 1).

### Association of Tau Variants With Clinical Outcome in Complex Injuries

**Figure 4.**
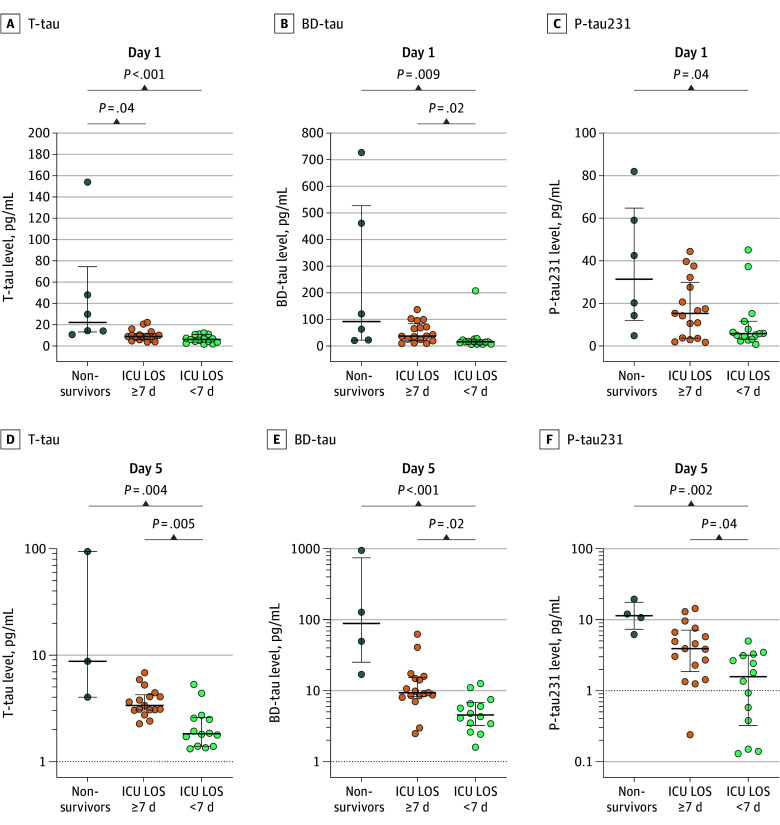
Tau Serum Levels According to Clinical Outcome Serum concentrations of total tau (t-tau), brain-derived tau (BD-tau), and phosphorylated tau 231 (p-tau231) from days 1 and 5 were stratified according to survival and intensive care unit (ICU) length of stay (LOS).

Finding biomarkers that inform on clinical outcome in the highly complex setting of multiple injuries is often difficult. We therefore sought to determine whether early and follow-up blood tau concentrations could be associated with the subsequent clinical course. Using t-tau as well as p-tau231 on day 1, there was only a significant difference between nonsurvivors and surviving patients (t-tau: 22 [IQR, 13-75] pg/mL for nonsurvivors vs 9 [IQR, 6-12] pg/mL for survivors with ICU LOS ≥ 7 d [*P* = .04] and 6 [IQR, 4-11] pg/mL for survivors with ICU LOS < 7 days [*P* < .001]; p-tau231: 31 [IQR, 12-65] pg/mL for nonsurvivors vs 6 [IQR, 3-12] pg/mL for survivors with ICU LOS < 7 days [*P* = .04]) (Figure 4A and C), while higher BD-tau serum concentrations were also associated with a worse clinical outcome (37 [IQR, 22-84] pg/mL for ICU LOS ≥ 7 days vs 16 [IQR, 7-23] pg/mL for ICU LOS < 7 days; *P* = .02) as reflected by the length of the stay in the ICU in surviving patients (Figure 4B). Interestingly, we found that this separation between the groups became clearer during the time in hospital. While there was hardly any difference in tau concentrations between outcome groups on arrival at the emergency department (eFigure 7 in Supplement 1), all 3 tau forms showed a significant difference among nonsurvivors, patients with complicated outcomes (ICU length of stay ≥7 days) and those with uncomplicated outcomes (ICU length of stay <7 days) on day 5 (t-tau: 2 [IQR, 1-3] pg/mL among survivors with uncomplicated vs 3 [IQR, 3-4] pg/mL with complicated outcomes [*P* = .005] and 9 [IQR, 4-94] pg/mL among nonsurvivors [*P* = .004]; BD-tau: 5 [IQR, 3-7] pg/mL among survivors with uncomplicated vs 9 [IQR, 8-15] pg/mL with complicated outcomes [*P* = .02] and 89 [IQR, 25-743] pg/mL among nonsurvivors [*P* < .001]; p-tau231: 2 [IQR, 0-3] pg/mL among survivors with uncomplicated vs 4 [IQR, 2-7] pg/mL with complicated outcomes [*P* = .04] and 11 [IQR, 7-18] pg/mL among nonsurvivors [*P* = .002]) (Figure 4D-F). We further assessed the discriminative potential of tau variants between groups when comparing patients with an uncomplicated outcome with patients with early deaths or longer ICU stays using receiver operating characteristics analysis. Herein, we found a good discrimination (area under the receiver operating characteristics curve >0.8), especially in tau levels on or after day 1 (eFigure 8 and eTable 14 in Supplement 1).

## Discussion

Tau forms as blood biomarkers in neurodegenerative diseases have been extensively studied in recent years.^4,10^ In addition to long-term neurodegeneration, Gonzalez-Ortiz et al^15^ recently demonstrated that TBI induces a pronounced release of BD-tau into the blood and correlates with poor outcome. However, studies in other acute settings such as cardiac arrest point to a possible role of other factors than direct traumatic neurological injury in tau release.^26,27^ To gain more insights into the intricate biology of tau posttranslational processing and release into circulation, we analyzed the recently described novel BD-tau form as well as t-tau and 2 p-tau forms in a well-characterized cohort of patients with multiple injuries.

The mechanisms behind elevated levels of tau in the blood of patients with multiple traumatic injuries are not clear but could be attributed to several interrelated mechanisms, reflecting both direct and indirect consequences of trauma. For instance, it is possible that severe multiple traumas can result in neuronal injury or death, leading to the release of intracellular tau proteins into the extracellular space and subsequently into the bloodstream, as also seen for other neuronal or astroglial proteins such as neurofilament light chain (NfL) or glial fibrillary acidic protein (GFAP).^28^ On the other hand, systemic effects of trauma also play an important role in tau elevation in blood. Hypoxia and ischemia, common consequences of multiple traumas due to substantial blood loss and hypovolemic shock, reduce oxygen supply to the brain, causing neuronal damage and promoting tau release into blood. Moreover, injury severity seems to play an important role in the blood tau levels. Patients with more severe injuries often experience greater neuronal damage and BBB disruption, resulting in higher blood tau levels and poorer clinical outcome. Different tau variants, including BD-tau and p-tau231, may exhibit distinct release patterns related to their brain origin and processing within neurons, hence the persistence of high BD-tau levels even 10 days after trauma.

In this study, we were able to show that blood levels of all tau forms were associated with the severity of the shock. Our findings indicate that secondary effects such as hypoxia due to severe hemorrhage may influence acute and delayed neuronal degeneration and the release of tau forms into the extracellular space in patients with multiple traumatic injuries. In addition, augmented systemic inflammation after bleeding^29^ could result in further disruption of the BBB, thereby facilitating enhanced release of tau forms into the circulation. Furthermore, increased injury and dysfunction of organs such as the kidney and the intestine may cause additional secondary neuronal injury.^21,30^ We also found higher levels of tau in the blood of patients with a more severe injury pattern, probably due to greater neuronal loss and disruption of the BBB. The same reason may apply to the association with GCS at the site of injury, where we found higher blood tau levels in patients with impaired consciousness. However, the observed lack of association between blood tau levels and the extent of brain damage measured by emergency imaging techniques as reflected in the AIS head score was unexpected. This was also the case when patients were grouped into those with and without intracranial hemorrhage. One possible interpretation of this finding is that the downstream consequences of shock may obscure or outweigh indicators typically associated with head trauma. For instance, neuronal changes related to shock (such as those linked to reduced oxygen supply) might present more prominently than those initially resulting from the head injury. Conversely, elevated levels of tau forms were also observed in the blood of patients with trauma but not TBI. This observation gives rise to the question of the source of these elevated blood levels; the systemic inflammation and significant disruption of the BBB in turn may lead to an increased leakage of tau forms present in the central nervous system into the bloodstream. Furthermore, peripheral sources of tau protein have been identified for both t-tau and p-tau^9,31,32^ and could be responsible for higher blood levels dependent on injury severity.

Recently, BD-tau has also been shown to hold strong prognostic value beyond trauma-related and degenerative conditions.^33^ In patients with Creutzfeldt-Jakob disease, blood BD-tau concentrations were markedly elevated and associated with disease progression and survival time.^33^ This finding underscores the robustness of BD-tau as a marker of acute and rapidly progressive neuronal degeneration, further supporting its potential as a universal indicator of neuroaxonal injury across diverse pathological contexts. The present data also indicate that the variants of tau correlate with clinical outcome. Elevated blood tau levels of all forms were observed as early as day 1 in nonsurviving patients compared with patients with trauma and a more favorable course. This difference was found to be even more pronounced at later time points, suggesting that the longer blood tau levels remain significantly elevated, the worse the outcome may be.

Other neurological biomarkers that have been studied in the blood of patients with trauma include NfL, GFAP, and beta-synuclein. NfL, a neuroaxonal marker, shows a completely different pattern over time after injury compared to the tau forms, with blood levels rising steadily over the first 10 days.^34^ At the same time, NfL and the different tau forms as shown by our data were correlated with a poor outcome in patients with trauma early after injury. In contrast, levels of GFAP, an astrocytic marker, and beta-synuclein, a synaptic marker, increase early after injury and decrease over time, similar to tau.^28^ However, compared with GFAP and beta-synuclein, tau appears to be the better marker of shock-induced neurological damage in the patient with multiple injuries.

Recent evidence has highlighted that plasma tau and p-tau levels can also be influenced by several nondegenerative factors. For instance, metabolic and systemic conditions such as elevated body mass index and impaired kidney function have been associated with altered plasma tau concentrations, likely due to differences in protein clearance and systemic inflammatory load.^35,36^ Furthermore, transient increases in plasma t-tau and p-tau levels have been reported following major cardiac surgery, showing a trajectory remarkably similar to that observed in our cohort.^37^ These findings suggest that systemic physiological stress and extracerebral factors may significantly modulate circulating tau levels, independent of direct neuronal injury.^37^

### Strengths and Limitations

The strengths of the present study include the longitudinal design, the clinically well characterized cohort of patients with severe multiple traumatic injuries and measurement of 4 different tau forms. As limitations, the lack of cerebrospinal fluid biomarker data, tau-positron emission tomographic images, and length of loss of consciousness and posttraumatic amnesia data of the patients have to be mentioned. Moreover, the nonsurvivor group was relatively small; no racial, ethnic, or socioeconomic status data were available; and the study lacks a validation cohort. These constraints reflect the challenges of recruiting severely injured patients under strict inclusion and exclusion criteria and within the acute phase of trauma care. Nonetheless, in our opinion, the data provide novel insights into the differential release of tau variants in this population. This study may thus represent a foundational step toward larger, multicenter investigations with long-term follow-up, including neurological outcome scores. This approach could provide more insights into the association of posttraumatic tau alterations with delayed neurodegeneration and provide biomarker tools to support clinical decision-making. Finally, in our study, the absence of detailed descriptors of therapeutic interventions (eg, vasopressor use, surgical procedures, or mechanical ventilation) represents an important limitation, as these factors could have affected biomarker kinetics. Likewise, the lack of available apolipoprotein E genotype information prevents assessment of potential genotype-specific differences in tau metabolism and clearance. Future studies should incorporate these parameters to better disentangle the relative contribution of neurological and systemic factors to plasma tau dynamics after severe injury.

## Conclusions

This cohort study highlights the potential value of blood-based tau biomarkers in understanding and monitoring neurological damage in patients with multiple traumatic injuries. Elevated levels of all tau variants—t-tau, BD-tau, and p-tau—were associated with injury severity, systemic shock, and poor clinical outcomes. In addition to central sources, it cannot be excluded that peripheral sources contributed to elevated blood levels of tau variants. Among these, BD-tau showed unique characteristics, with sustained elevation beyond 10 days. This suggests that the design of the BD-tau assay, along with the observed pattern of sustained elevation after the initial phase of general tau biomarker increase, renders this particular biomarker particularly useful to quantify neuronal injury sustained in multiple-trauma and underscores its potential as a robust marker for ongoing neuronal injury. The data also indicate that in complex injury settings, markers become increasingly reliable in their association with outcome after the first few days.

The ability of tau markers to distinguish between patients with different injury severities and to link with outcomes early after trauma makes promising monitoring tools in both clinical and research settings. Furthermore, their sensitivity to systemic factors, such as hypoxia and inflammation, suggests that tau biomarkers are not solely reflective of direct brain injury but may also capture the broader systemic consequences of trauma. This wide applicability could complement traditional assessments of patients with acute neurological damage.

## Notes

Supplement 1. **eMethods.** Detailed Method Descriptions **eTable 1.** Characterization of Head Injury Patterns in the Polytrauma Cohort **eFigure 1.** Correlation of Tau With Age **eTable 2.** Correlation of Tau Biomarkers With Age in the Control Group **eTable 3.** Correlation of Tau Biomarkers With Age in the Polytrauma Cohort **eFigure 2.** Differences in Tau Form Concentrations Stratified by Sex **eTable 4.** Median and IQR of the Tau Variants in Polytrauma and Control Groups **eTable 5.** BD-Tau After Adjustment for Repeated Measures, Age, and Sex **eTable 6.** T-Tau After Adjustment for Repeated Measures, Age, and Sex **eTable 7.** P-Tau231 After Adjustment for Repeated Measures, Age, and Sex **eFigure 3.** P-Tau217 Trajectory in Multiply Injured Patients **eFigure 4.** Correlations Between Tau Forms **eFigure 5.** Serum Tau Levels According to Severity of Head Injury and Overall Injury **eTable 8.** BD-Tau Group Statistics Over Time After Adjustment for Sex and Age **eTable 9.** Contrast Between BF-Tau Groups as Estimate of Emmean Differences **eTable 10.** T-Tau Group Statistics Over Time After Adjustment for Sex and Age **eTable 11.** Contrast Between T-Tau Groups as Estimate of Emmean Differences **eTable 12.** P-Tau231 Group Statistics Over Time After Adjustment for Sex and Age **eTable 13.** Contrast Between P-Tau231 Groups as Estimate of Emmean Differences **eFigure 6.** Tau Correlations With Shock Parameters at Day 1 **eFigure 7.** Tau Serum Levels at Day 0 According to Outcome **eFigure 8.** ROC Analyses of the 3 Tau Forms According to Clinical Outcome **eTable 14.** Number of Patients for ROC Analysis

Supplement 2.
Data Sharing Statement
