# Supplementary material for: Blood-Based Analysis of Different Tau Variants in Patients With Multiple Traumatic Injuries
Source: JAMA Netw Open. 2026 Feb 10;9(2):e2558573. doi: 10.1001/jamanetworkopen.2025.58573 (PMC12892155; doi:10.1001/jamanetworkopen.2025.58573)
Supplement: Supplement 1. — eMethods. Detailed Method Descriptions eTable 1. Characterization of Head Injury Patterns in the Polytrauma Cohort eFigure 1. Correlation of Tau With Age eTable 2. Correlation of Tau Biomarkers With Age in the Control Group eTable 3. Correlation of Tau Biomarkers With Age in the Polytrauma Cohort eFigure 2. Differences in Tau Form Concentrations Stratified by Sex eTable 4. Median and IQR of the Tau Variants in Polytrauma and Control Groups eTable 5. BD-Tau After Adjustment for Repeated Measures, Age, and Sex eTable 6. T-Tau After Adjustment for Repeated Measures, Age, and Sex eTable 7. P-Tau231 After Adjustment for Repeated Measures, Age, and Sex eFigure 3. P-Tau217 Trajectory in Multiply Injured Patients eFigure 4. Correlations Between Tau Forms eFigure 5. Serum Tau Levels According to Severity of Head Injury and Overall Injury eTable 8. BD-Tau Group Statistics Over Time After Adjustment for Sex and Age eTable 9. Contrast Between BF-Tau Groups as Estimate of Emmean Differences eTable 10. T-Tau Group Statistics Over Time After Adjustment for Sex and Age eTable 11. Contrast Between T-Tau Groups as Estimate of Emmean Differences eTable 12. P-Tau231 Group Statistics Over Time After Adjustment for Sex and Age eTable 13. Contrast Between P-Tau231 Groups as Estimate of Emmean Differences eFigure 6. Tau Correlations With Shock Parameters at Day 1 eFigure 7. Tau Serum Levels at Day 0 According to Outcome eFigure 8. ROC Analyses of the 3 Tau Forms According to Clinical Outcome eTable 14. Number of Patients for ROC Analysis [file jamanetwopen-e2558573-s001.pdf]

Supplementary Online Content

Halbgebauer R, Gonzalez-Ortiz F, Mayer B, et al. Blood-based analysis of different tau variants in patients with multiple traumatic injuries. *JAMA Netw Open.* 2026;9(2):e2558573. doi:10.1001/jamanetworkopen.2025.58573

eMethods. Detailed Method Descriptions
eTable 1. Characterization of Head Injury Patterns in the Polytrauma Cohort
eFigure 1. Correlation of Tau With Age
eTable 2. Correlation of Tau Biomarkers With Age in the Control Group
eTable 3. Correlation of Tau Biomarkers With Age in the Polytrauma Cohort
eFigure 2. Differences in Tau Form Concentrations Stratified by Sex
eTable 4. Median and IQR of the Tau Variants in Polytrauma and Control Groups
eTable 5. BD-Tau After Adjustment for Repeated Measures, Age, and Sex
eTable 6. T-Tau After Adjustment for Repeated Measures, Age, and Sex
eTable 7. P-Tau231 After Adjustment for Repeated Measures, Age, and Sex
eFigure 3. P-Tau217 Trajectory in Multiply Injured Patients
eFigure 4. Correlations Between Tau Forms
eFigure 5. Serum Tau Levels According to Severity of Head Injury and Overall Injury
eTable 8. BD-Tau Group Statistics Over Time After Adjustment for Sex and Age
eTable 9. Contrast Between BF-Tau Groups as Estimate of Emmean Differences
Table 10. T-Tau Group Statistics Over Time After Adjustment for Sex and Age
eTable 11. Contrast Between T-Tau Groups as Estimate of Emmean Differences
eTable 12. P-Tau231 Group Statistics Over Time After Adjustment for Sex and Age
eTable 13. Contrast Between P-Tau231 Groups as Estimate of Emmean Differences
eFigure 6. Tau Correlations With Shock Parameters at Day 1
eFigure 7. Tau Serum Levels at Day 0 According to Outcome
eFigure 8. ROC Analyses of the 3 Tau Forms According to Clinical Outcome
eTable 14. Number of Patients for ROC Analysis
eReferences.

This supplementary material has been provided by the authors to give readers additional information about their work.

© 2026 Halbgebauer R et al. *JAMA Network Open*.

eMethods. Detailed Method Descriptions

Clinical study

A monocenter, controlled, longitudinal observational study with prospective sample collection was conducted according to the Declaration of Helsinki and its modifications. The study protocol was approved by the Local Ethics Committee of Ulm University, and the study is still ongoing (approvals #244/11, #94/14, #260/22). Study participants were recruited starting from December 2013 at Ulm University Hospital (Level I Trauma Care Center) after informed written consent; if patients were unconscious at the time of hospital admission, consent was obtained after they regained consciousness or from their legal representatives. Polytrauma patients were enrolled in the study after clinical examination and whole-body computed tomography if their Injury Severity Score (ISS)[1] in the emergency room was 18 or higher according to the “Berlin definition” of polytrauma with at least two AIS scores of 3 or more[2]. Serum was collected upon hospital admission and 1 d, 5 d, and 10 d after admission. Samples were stored at -80°C until analysis in November 2022. Clinical data and imaging data were collected retrospectively from the records. Patients were followed up in regards to their clinical course (*i.e.*, survival, length of stay on the intensive care unit (ICU), length of stay in hospital) until hospital discharge. Polytrauma patients were classified into suffering from TBI when the Abbreviated Injury Scale score of the Head (AISH) (ranging from 0 = no injury at all, to 6 = fatal injury)[3,4] was 2 or higher depending on computed-tomography analysis at the emergency department. Only 2 patients in the AISH ≥ 2 group had no intracranial hemorrhage. All patients in the minor head injury group had no intracranial hemorrhage (for further head injury data on the whole polytrauma cohort, see supplementary **eTable 1**). The AISH was used because the Glasgow Coma Scale (GCS) may be less reliable in the presence of sedation at the time of hospital admission as commonly seen in multiply injured patients. However, we also evaluated the GCS score which has the advantage that it can be obtained by the emergency physician at the scene. Patients were classified into three groups according to their state of consciousness according to their GCS values at the site of injury: severe (GCS 3-8), moderate (GCS 9-12), and mild (GCS 13-15)[5,6]. In addition, patients were stratified according to the presence or absence of hemorrhagic shock based on initial base excess (BE) < −6

© 2026 Halbgebauer R et al. *JAMA Network Open*.

mmol/l or lactate ≥ 2.5 mmol/l (i.e. as determined upon arrival at the emergency department), or red blood cell (RBC) concentrates transfused during the first 24 h after admission > 2 U[7,8]. Furthermore, as a surrogate parameter for clinical outcome, we grouped patients into non-survivors, patients with a complicated clinical course (with a length of stay on the ICU of 7 or more days), and those with an uncomplicated clinical course (length of stay on the ICU less than 7 days).

Potential participants for the healthy volunteers group (n=24) were recruited at Ulm University by internal communication channels such as staff newsletters, bulletin boards, and institutional mailing lists. None of the healthy volunteers reported to have a history of any neurological condition.

Serum biomarker measurements

Serum BD-tau, p-tau231, and p-tau217 measurements were performed on the Simoa HD-X platform (Quanterix, MA, USA) with a method previously described[9,10]. Serum t-tau was measured on the Simoa HD-X using the Quanterix kit (#101552). Quality control samples were analyzed in duplicates at the start and the end of each plate to assess precision. Intraplate variation was determined with internal quality controls (iQC) at the beginning and end of the plate (CV<10% for all the markers). Intermediate precision was determined with iQC at the beginning of each plate with the following results: BD-tau %CV: 5.4%; p-tau231 %CV: 8.4%; total tau %CV: 9.6% and p-tau217 %CV: 9.7%. For each assay, identical batches of reagents were used for all matrix types to enable comparison of results. These measurements were performed at the Clinical Neurochemistry Laboratory, Sahlgrenska University Hospital, Mölndal, Sweden. P-tau217 measurements were only performed in n = 18 patients and no healthy controls due to limited sample volumes, and p-tau217 was thus excluded from in-depth statistical and clinical analysis.

© 2026 Halbgebauer R et al. *JAMA Network Open*.

Statistical analysis

Data are shown as individual values with median and interquartile ranges. Normal distribution of tau biomarkers was assessed with the Shapiro-Wilk test, and since the results indicated that the data were not normally distributed, non-parametric tests were employed for subsequent analyses. Comparisons between three or more groups were performed using Kruskal-Wallis analysis followed by Dunn's correction for multiple comparisons in non-normally distributed data sets. The polytrauma patients stratified for clinical parameters were compared for the respective time point by means of Mann-Whitney U testing in case of two groups and Kruskal-Wallis analysis with Dunn's post-hoc correction for three groups. Sex comparison was done employing Fisher's exact test, and age was compared using Mann-Whitney U testing. Receiver-operating curve (ROC) analysis was performed to assess the ability of tau measurements at different time points (at day 0, 1, 5, 10) to discriminate between uncomplicated outcome (ICU stay < 7 days) and complicated outcome (i.e. early death or ICU stay ≥ 7 days). Therefore, the area under the curve (AUC) and corresponding 95% confidence intervals was calculated. Correlation analyses were performed using spearman rank test. These analyses were performed using Prism (v. 10.4.0, GraphPad). To verify univariate findings for biomarker analysis, age- and sex-adjusted estimates of group differences were calculated using mixed linear regression modelling (*lmer* and *emmeans* packages in R) using linear contrast hypotheses. Specifically, these models used BD-tau/t-tau/p-tau231 as the outcome variable, respectively. Age, sex, group status, time, as well as the interaction of group status and time served as the fixed model parameters. A random intercept was used to account for the repeated measurement structure of these data. For this approach, we employed the R software for statistical computing (v. 4.5.0). The null hypothesis of no inter-group differences was rejected for p-values < 0.05 in two-sided tests.

© 2026 Halbgebauer R et al. *JAMA Network Open*.

Additional clinical information on the polytrauma cohort

eTable 1. Characterization of Head Injury Patterns in the Polytrauma Cohort

	Number of polytrauma patients
Intracranial hemorrhage yes/no	25/20
Intracranial surgery	9/36
Intubated by ER clinician	28/17

Only 2 patients with TBI according to the AISH (AISH ≥ 2) had no intracranial hemorrhage. All patients with no TBI according to the AISH also had no intracranial hemorrhage. When grouping the polytrauma patients according to intracranial hemorrhage, the results therefore were only marginally different from the classification using the AISH (data not shown). We also analyzed the stratification according to intracranial surgery (yes or no). We did not detect a significant difference in BD-tau, p-tau and t-tau comparing groups at any time point. The same was true when stratified according to intubation by the ER clinician (data not shown).

eFigure 1. Correlation of Tau With Age

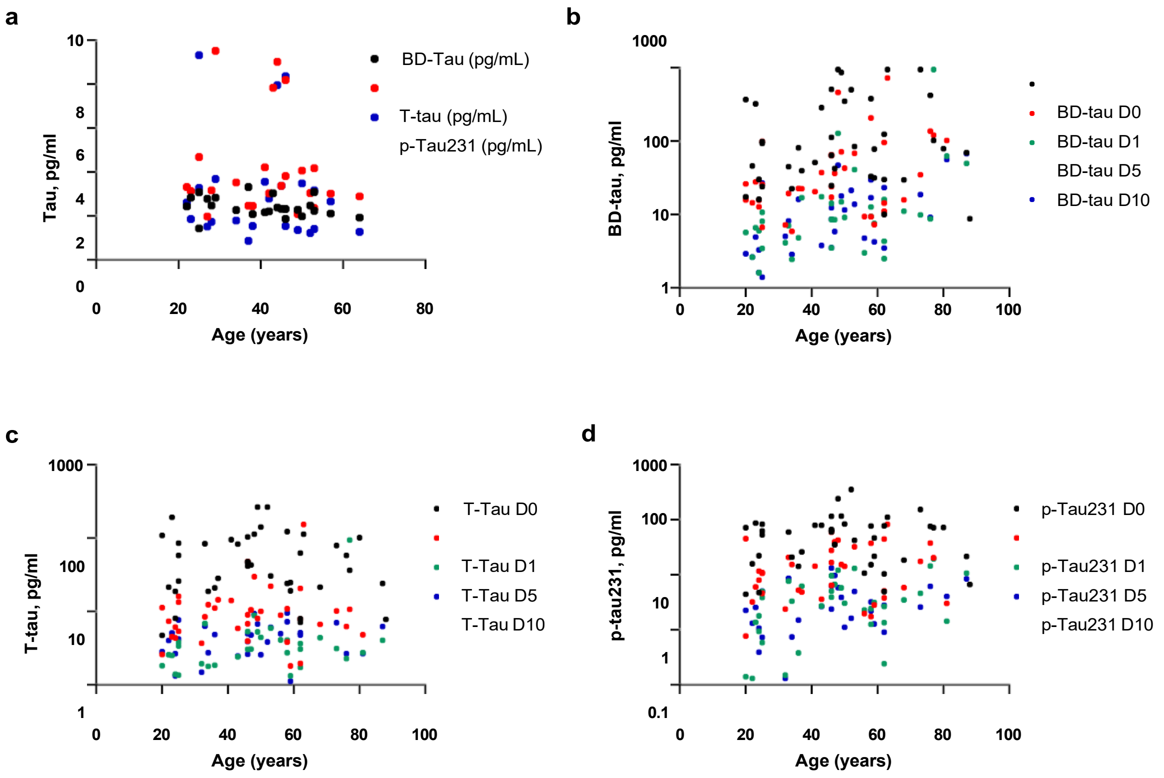

Figure A displays the healthy volunteer group with no detected association between age and any of the tau forms. Figure B-C illustrates the correlation of the different tau forms with age in the polytrauma cohort. Weak to moderate correlations were found for BD-tau at d1, 5 and 10 as well as for t-tau and p-tau231 at d5.
Abbreviations: BD-tau, brain-derived tau; p-tau231, phosphorylated tau 231; t-tau, total tau.

© 2026 Halbgebauer R et al. *JAMA Network Open*.

eTable 2. Correlation of Tau Biomarkers With Age in the Control Group

Tau Biomarker	Spearman r and 95% CI	p-value
BD-tau	-0.33 (-0.65-0.10)	0.12
T-tau	-0.2 (-0.57-0.23	0.34
p-tau231	-0.18 (-0.56-0.25)	0.39

eTable 3. Correlation of Tau Biomarkers With Age in the Polytrauma Cohort

Tau Biomarker and day of sampling	Spearman r and 95% CI	p-value
BD-tau, d0	0.15 (-0.18-0.45)	0.36
BD-tau, d1	0.37 (0.05-0.62	0.02
BD-tau, d5	0.51 (0.20-0.72)	0.02
BD-tau, d10	0.61 (0.32-0.80)	0.0003
T-tau, d0	-0.00 (-0.33-0.31)	0.96
T-tau, d1	0.06 (-0.27-0.38)	0.70
T-tau, d5	0.35 (0.00-0.62)	0.04
T-tau, d10	0.22 (-0.16-0.54)	0.24
p-tau231, d0	0.05 (-0.26-0.36)	0.73
p-tau231, d1	0.17 (-0.17-0.47)	0.30
p-tau231, d5	0.38 (0.04-0.64)	0.03
p-tau231, d10	0.33 (-0.05-0.62)	0.08

© 2026 Halbgebauer R et al. *JAMA Network Open*.

eFigure 2. Differences in Tau Form Concentrations Stratified by Sex

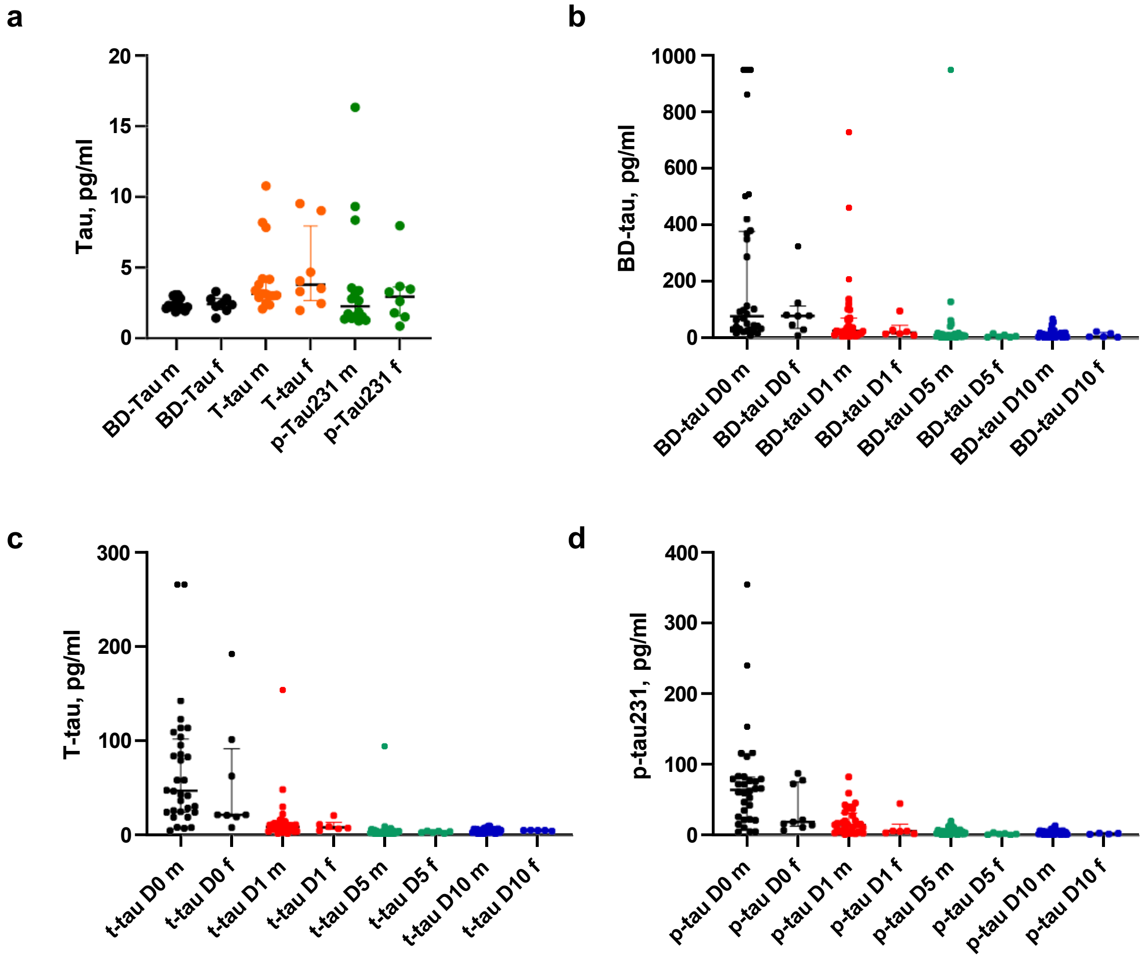

Figure A shows the thre tau biomarkers in the healthy volunteers grouped by sex. No difference between male and females was detected. Figure B-D display the tau concentrations in the polytrauma cohort according to sex and sampling date. No significant differences were found. Shown are the median and inter-quartile ranges. Abbreviations: BD-tau, brain-derived tau; p-tau231, phosphorylated tau 231; t-tau, total tau.

© 2026 Halbgebauer R et al. *JAMA Network Open*.

eTable 4. Median and IQR of the Tau Variants in Polytrauma and Control Groups

**BD-tau**	Day 0	Day 1	Day 5	Day 10	Controls
25% Percentile	30.40	14.39	4.310	3.510	2.115
Median	78.16	24.71	8.550	8.360	2.315
75% Percentile	342.6	69.08	14.81	18.04	2.818
**T-tau**					
25% Percentile	21.38	5.210	1.910	2.650	2.920
Median	42.98	8.665	3.070	4.760	3.340
75% Percentile	95.00	12.58	4.158	6.230	4.563
**p-tau231**					
25% Percentile	20.99	4.775	1.340	1.300	1.428
Median	61.40	11.28	3.120	2.565	1.820
75% Percentile	79.30	28.77	6.200	5.248	3.243

eTable 5. BD-Tau After Adjustment for Repeated Measures, Age, and Sex

Group	Emmean	SE	Lower CL	Upper CL
Healthy	3.0887	35.0	-66.07	72.2
PT d0	187.3251*	28.6	130.72	243.9
PT d1	51.2519	29.8	-7.65	110.2
PT d5	29.2958	30.6	-31.26	89.9
PT d10	0.0184	32.2	-63.62	63.7

*p<0.05 compared to Healthy. Emmean, estimated marginal mean. SE, standard error. CL, confidence limit.

eTable 6. T-Tau After Adjustment for Repeated Measures, Age, and Sex

Group	Emmean	SE	Lower CL	Upper CL
Healthy	3.77	7.23	-10.52	18.1
PT d0	64.62*	5.90	52.97	76.3
PT d1	12.50	6.14	0.36	24.6
PT d5	5.41	6.39	-7.23	18.0
PT d10	3.42	6.68	-9.78	16.6

*p<0.05 compared to Healthy. Emmean, estimated marginal mean. SE, standard error. CL, confidence limit.

eTable 7. P-Tau231 After Adjustment for Repeated Measures, Age, and Sex

Group	Emmean	SE	Lower CL	Upper CL
Healthy	1.63	8.01	-14.26	17.5
PT d0	59.80*	6.50	46.88	72.7
PT d1	15.81	6.75	2.43	29.2
PT d5	5.29	6.88	-8.35	18.9
PT d10	1.53	7.19	-12.70	15.8

*p<0.05 compared to Healthy. Emmean, estimated marginal mean. SE, standard error. CL, confidence limit.

© 2026 Halbgebauer R et al. *JAMA Network Open*.

eFigure 3. P-Tau217 Trajectory in Multiply Injured Patients

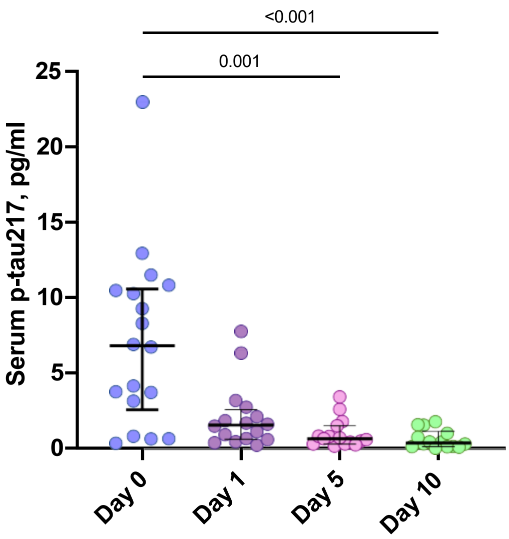

p-tau217 was measured in serum samples taken from polytrauma patients upon arrival at the emergency department (Day 0) and at the designated time points after injury. Abbreviation: P-tau217, phosphorylated tau 217.

© 2026 Halbgebauer R et al. *JAMA Network Open*.

eFigure 4. Correlations Between Tau Forms

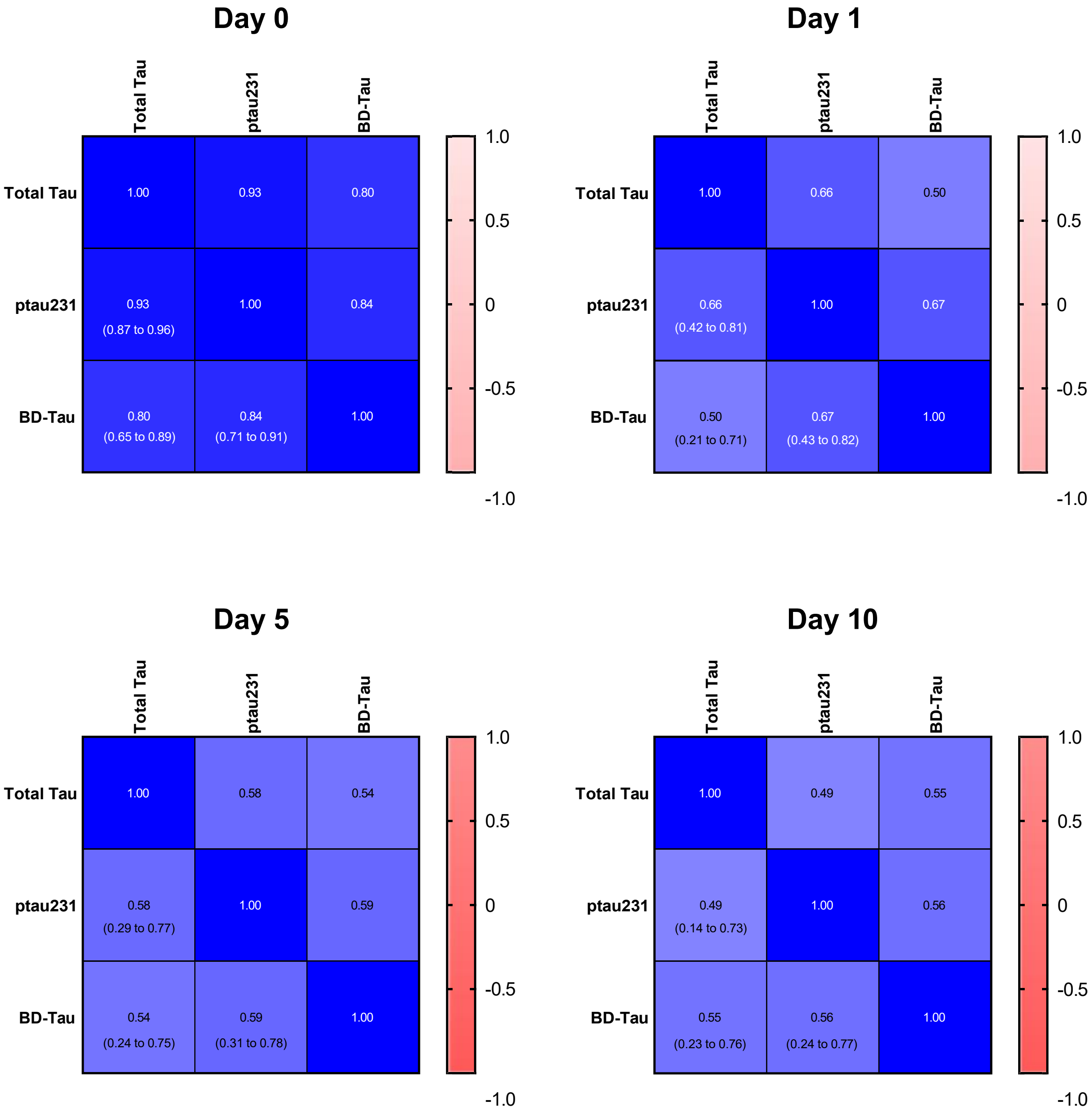

Spearman coefficients for the correlation between plasma concentrations of the tau forms at respective timepoints. The 95% confidence interval is given in brackets.

© 2026 Halbgebauer R et al. *JAMA Network Open*.

eFigure 5. Serum Tau Levels According to Severity of Head Injury and Overall Injury

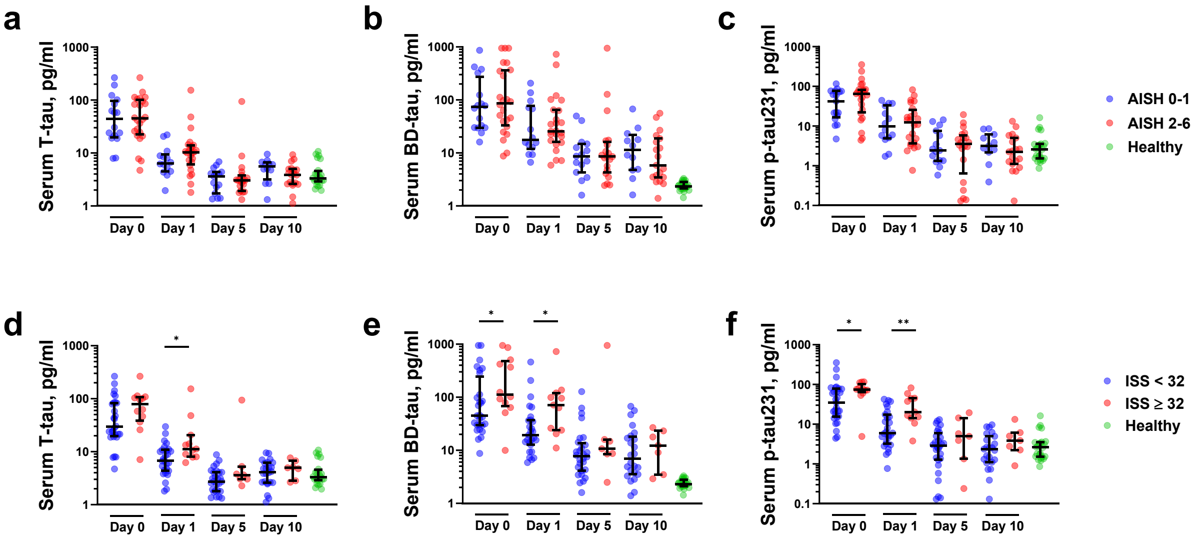

(a-c) The severity of head injury was assessed according to the Abbreviated injury scale of the head (AISH) using emergency room computed tomography analysis, stratifying patients into those with no or minor head injury (AISH 0-1) or those with significant head injury (AISH 2-6). Serum levels of total tau (a), BD-tau (b), and p-tau231 (c) during the observation period are shown for patients with and without TBI with controls for reference. (d-f) For overall injury severity, patients were assessed for the total Injury severity score (ISS) and stratified into patients with moderate overall injury severity (ISS < 32) or severe overall injury (ISS ≥ 32). Serum levels of total tau (d), BD-tau (e), and p-tau231 (f) during the observation period for moderately injured patients and severely injured patients with healthy volunteers as a reference are shown. Data for healthy volunteers is shown twice for the respective parameters. Patient groups at respective time points were compared using Mann-Whitney testing.

© 2026 Halbgebauer R et al. *JAMA Network Open*.

eTable 8. BD-Tau Group Statistics Over Time After Adjustment for Sex and Age

Group means for TBI (1) and no TBI (0)

Time [d]	TBI Group	Emmean	SE	Lower CL	Upper CL
0	0	140.81	46.6	48.5	233.1
0	1	211.67	42.1	128.1	295.3
1	0	28.22	49.6	-69.9	126.4
1	1	59.62	43.0	-25.7	144.9
5	0	-12.61	48.4	-108.4	83.2
5	1	54.96	45.5	-35.3	145.3
10	0	-17.76	53.2	-123.0	87.5
10	1	6.71	46.3	-85.1	98.6

eTable 9. Contrast Between BD-Tau Groups as Estimate of Emmean Differences

Time	Contrast	Estimate	SE	p-value
0	TBI0 - TBI1	-70.9	60.0	0.2400
1	TBI0 - TBI1	-31.4	62.1	0.6139
5	TBI0 - TBI1	-67.6	62.9	0.2847
10	TBI0 - TBI1	-24.5	67.1	0.7160

Emmean, estimated marginal mean. SE, standard error. CL, confidence limit.

eTable 10. T-Tau Group Statistics Over Time After Adjustment for Sex and Age

Group means for TBI (1) and no TBI (0)

Time	TBI Group	Emmean	SE	Lower CL	Upper CL
0	0	68.63	9.45	49.92	87.30
0	1	64.82	7.71	49.56	80.10
1	0	7.78	10.10	-12.19	27.70
1	1	18.60	7.72	3.32	33.90
5	0	3.22	9.77	-16.11	22.60
5	1	11.65	8.64	-5.44	28.70
10	0	3.52	10.90	-17.96	25.00
10	1	7.29	8.64	-9.80	24.40

eTable 11. Contrast Between T-Tau Groups as Estimate of Emmean Differences

Time	Contrast	Estimate	SE	p-value
0	TBI0 - TBI1	3.80	12.2	0.7558
1	TBI0 - TBI1	-10.82	12.7	0.3961
5	TBI0 - TBI1	-8.43	13.0	0.5193
10	TBI0 - TBI1	-3.77	13.9	0.7863

Emmean, estimated marginal mean. SE, standard error. CL, confidence limit.

© 2026 Halbgebauer R et al. *JAMA Network Open*.

eTable 12. P-Tau231 Group Statistics Over Time After Adjustment for Sex and Age
Group means for TBI (1) and no TBI (0)

Time	TBI Group	Emmean	SE	Lower CL	Upper CL
0	0	42.725	11.5	19.72	65.70
0	1	68.575	11.0	46.54	90.60
1	0	12.167	12.0	-11.84	36.20
1	1	17.096	11.1	-5.19	39.40
5	0	0.334	11.9	-23.44	24.10
5	1	7.434	11.4	-15.37	30.20
10	0	-4.286	12.7	-29.56	21.00
10	1	4.047	11.5	-18.92	27.00

eTable 13. Contrast Between P-Tau231 Groups as Estimate of Emmean Differences

Time	Contrast	Estimate	SE	p-value
0	TBI0 - TBI1	-25.85	14.9	0.0873
1	TBI0 - TBI1	-4.93	15.2	0.7471
5	TBI0 - TBI1	-7.10	15.3	0.6445
10	TBI0 - TBI1	-8.33	15.9	0.6027

Emmean, estimated marginal mean. SE, standard error. CL, confidence limit.

© 2026 Halbgebauer R et al. *JAMA Network Open*.

eFigure 6. Tau Correlations With Shock Parameters at Day 1

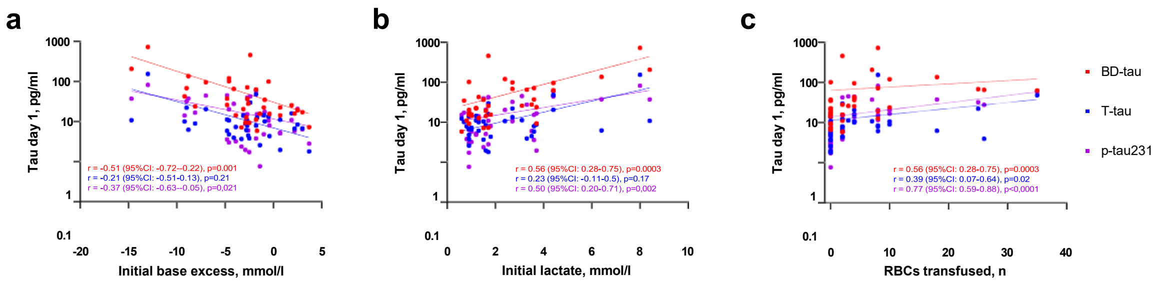

(a-c) Correlations of the tau markers at day 1 with shock parameters. Tau forms partially displayed a correlation to initial base excess and lactate as well as the number of RBCs transfused during the first 24 h. Abbreviations: RBCs, red blood cell concentrates; BD-tau, brain-derived tau; p-tau231, phosphorylated tau 231; t-tau, total tau. Spearman correlation coefficient r is shown with its 95% confidence interval.

© 2026 Halbgebauer R et al. *JAMA Network Open*.

eFigure 7. Tau Serum Levels at Day 0 According to Outcome

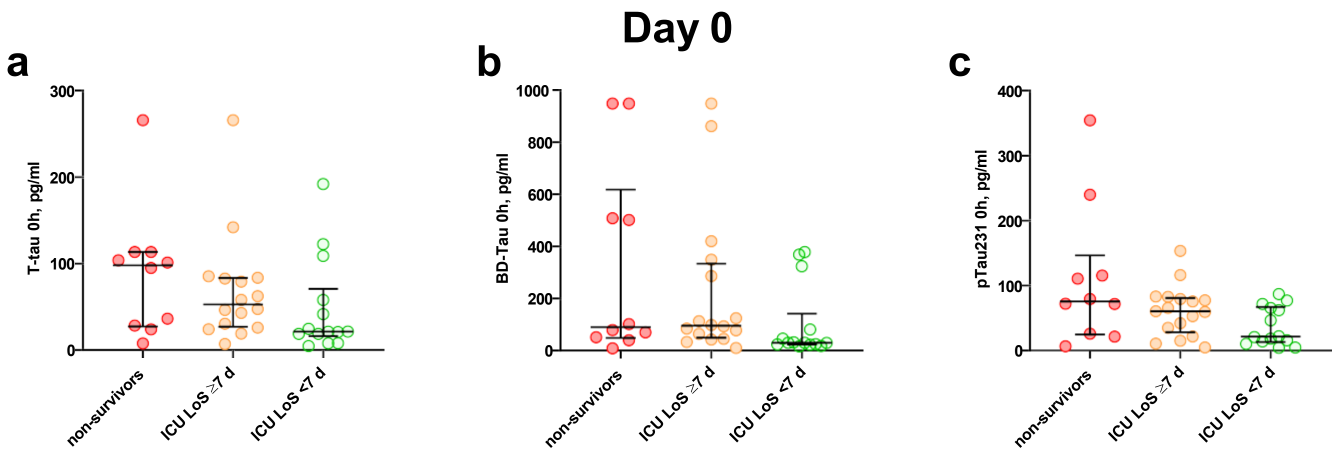

Patients were stratified into those who died during the observation period of 10 days post admission, and survivors with a complicated outcome (length of stay (LoS) in the ICU ≥ 7
d) and those with an uncomplicated outcome (LoS in the ICU < 7 d). Groups were compared using Kruskal-Wallis testing, with no significant inter-group differences.

eFigure 8. ROC Analyses of the 3 Tau Forms According to Clinical Outcome

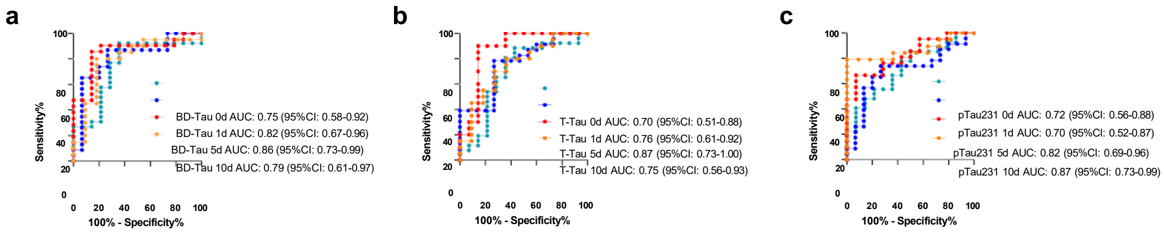

Patients were grouped into uncomplicated outcome (Stay in the ICU <7 days) and into those with early deaths or longer ICU stays (≥7 days in the ICU). **a** depicts the BD-Tau results for the four different time points showing the highest AUC at day 5. **b** illustrates the t-Tau findings with the highest AUC also at day 5. In **c** the p-tau231 are shown with an even higher AUC at day 10 compared to day 5. Number of patients can be found in table S4. Abbreviations: BD-tau, brain-derived tau; CI, confidence interval; p-tau231, phosphorylated tau 231; t-tau, total tau.

eTable 14. Number of Patients for ROC Analysis

	n stay in ICU < 7days	n stay in ICU ≥ 7 day + non-survivors
BD-Tau d0	14	26
BD-Tau d1	15	23
BD-Tau d5	14	21
BD-Tau d10	11	20
T-Tau d0	14	26
T-Tau d1	15	23
T-Tau d5	14	20
T-Tau d10	11	20
p-Tau231 d0	14	27
p-Tau231 d1	25	23
p-Tau231 d5	14	21
p-Tau231 d10	11	19

© 2026 Halbgebauer R et al. *JAMA Network Open*.

eReferences.

1. Greenspan L, McLellan BA, Greig H. Abbreviated Injury Scale and Injury Severity Score: a scoring chart. *J Trauma*. 1985;25(1):60-64. doi:10.1097/00005373-198501000-00010

2. Pape HC, Lefering R, Butcher N, et al. The definition of polytrauma revisited: An international consensus process and proposal of the new "Berlin definition." *J Trauma Acute Care Surg*. 2014;77(5):780-786. doi:10.1097/TA.0000000000000453

3. Paget LM, Boutonnet M, Moyer JD, et al. Trauma centre admissions for traumatic brain injury in France: One-year epidemiological analysis of prospectively collected data. *Anaesth Crit Care Pain Med*. 2021;40(1):100804. doi:10.1016/j.accpm.2021.100804

4. Camarano JG, Ratliff HT, Korst GS, Hrushka JM, Jupiter DC. Predicting in-hospital mortality after traumatic brain injury: External validation of CRASH-basic and IMPACT-core in the national trauma data bank. *Injury*. 2021;52(2):147-153. doi:10.1016/j.injury.2020.10.051

5. Teasdale G, Jennett B. Assessment of coma and impaired consciousness. A practical scale. *Lancet*. 1974;2(7872):81-84. doi:10.1016/s0140-6736(74)91639-0

6. Jain S, Iverson LM. Glasgow Coma Scale. In: *StatPearls*. StatPearls Publishing; 2025. Accessed January 15, 2025. http://www.ncbi.nlm.nih.gov/books/NBK513298/

7. Kruse O, Grunnet N, Barfod C. Blood lactate as a predictor for in-hospital mortality in patients admitted acutely to hospital: a systematic review. *Scand J Trauma Resusc Emerg Med*. 2011;19:74. doi:10.1186/1757-7241-19-74

8. Minei JP, Cuschieri J, Sperry J, et al. The changing pattern and implications of multiple organ failure after blunt injury with hemorrhagic shock. *Crit Care Med*. 2012;40(4):1129-1135. doi:10.1097/CCM.0b013e3182376e9f

9. Gonzalez-Ortiz F, Turton M, Kac PR, et al. Brain-derived tau: a novel blood-based biomarker for Alzheimer's disease-type neurodegeneration. *Brain*. 2023;146(3):1152-1165. doi:10.1093/brain/awac407

10. Ashton NJ, Pascoal TA, Karikari TK, et al. Plasma p-tau231: a new biomarker for incipient Alzheimer's disease pathology. *Acta Neuropathol*. 2021;141(5):709-724. doi:10.1007/s00401-021-02275-6

© 2026 Halbgebauer R et al. *JAMA Network Open*.
