# Supplementary material for: Blood-Based Analysis of Different Tau Variants in Patients With Multiple Traumatic Injuries
Source: JAMA Netw Open. 2026 Feb 10;9(2):e2558573. doi: 10.1001/jamanetworkopen.2025.58573 (PMC12892155; doi:10.1001/jamanetworkopen.2025.58573)
Supplement: Supplement 2. — Data Sharing Statement [file jamanetwopen-e2558573-s002.pdf]

Data Sharing Statement

Halbgebauer. Blood-Based Analysis of Different Tau Variants in Patients With Multiple Traumatic Injuries. *JAMA Netw Open*. Published February 10, 2026.
doi:10.1001/jamanetworkopen.2025.58573

Data
Data available: No
